# Influence of microsurgical decompression on segmental stability of the lumbar spine – One-year results in a prospective, consecutive case series using upright, kinetic-positional MRI

**DOI:** 10.1186/s12891-022-05701-2

**Published:** 2022-08-03

**Authors:** Dorothea Daentzer, Elina Venjakob, Jessica Schulz, Thorsten Schulze, Michael Schwarze

**Affiliations:** 1 Orthopedic Department, Hannover Medical School, DIAKOVERE Annastift, Anna-von-Borries-Str. 1-7, 30625 Hannover, Germany; 2 Privatpraxis für Upright Kernspintomographie Hannover, Expo-Plaza 10, 30539 Hannover, Germany; 3 Orthopedic Department, Laboratory for Biomechanics and Biomaterials, Hannover Medical School, Anna-von-Borries-Str. 1-7, 30625 Hannover, Germany

**Keywords:** Intersegmental angle, Kinetic positional MRI, Lumbar spine, Microsurgical decompression, Segmental stability, Upright-MRI

## Abstract

**Background:**

Standard procedure in patients with lumbar spinal canal stenosis is decompression to relieve the neural structures. Clinical results generally show superiority compared to nonoperative therapy after an observation period of several years. However, there is still a question of postsurgical segmental stability and correlation to clinical findings. Therefore, the aim of this prospective study was to evaluate the clinical outcome in patients who underwent microsurgical decompression in lumbar spine and particularly to analyze intervertebral movement by use of upright, kinetic-positional magnetic resonance imaging (MRI) over a period of 12 months and then to correlate the clinical and imaging data with each other.

**Methods:**

Complete clinical data of 24 consecutive participants with microsurgical decompression of the lumbar spine were obtained by questionnaires including visual analogue scale (VAS) for back and leg, Oswestry Disability Index (ODI), Roland-Morris Disability Questionnaire (RMDQ), Short-Form-36 (SF-36), walking distance and use of analgesics with assessment preoperatively and after 6 weeks and 12 months. At the same points of time all patients underwent upright, kinetic-positional MRI to measure intersegmental motion of the operated levels with determination of intervertebral angles and translation and to correlate the clinical and imaging data with each other.

**Results:**

VAS for leg, ODI, RMDQ and physical component scale of SF-36 improved statistically significantly without statistically significant differences regarding intersegmental motion and horizontal displacement 6 weeks and 12 months after operation. Regression analysis did not find any linear dependencies between the clinical scores and imaging parameters.

**Conclusions:**

In awareness of some limitations of the study, our results demonstrate no increase of intersegmental movement or even instability after microsurgical decompression of the lumbar spine over a follow-up period of 12 months, which is equivalent to preservation of intervertebral stability. Furthermore, the magnitude of intervertebral range of motion showed no correlation to the clinical score parameters at all three examination points of time.

## Background

Patients with evidence of spinal canal stenosis of the lumbar spine and similarly with a disk prolapse are potential candidates for surgical intervention, especially when their symptoms are resistant to conservative treatment or relevant neurological deficits exist. Decompression procedure is the therapy of choice with the aim to take the compressive elements away from the neural structures and to remove any herniated disk material out of the vertebral canal. According to the multicentric randomized “SPORT”-study (*Spine Patient Outcomes Research Trial*) the clinical results showed clear superiority of surgery for lumbar spinal canal stenosis and disc prolapse compared to nonoperative therapy after an observation period of four years [1].

In addition to the beneficial clinical findings it seems to be relevant to consider the postsurgical stability of the operated segments because of the necessity of removal of important posterior spinal elements during decompression like parts of laminae, facets and ligamentum flavum or disk material. Therefore, it can be assumed that the bigger the defect of the dorsal structures is, the more intense the intervertebral instability becomes.

Just limited data from experimental research exist supporting this theory [2–4]. Of course, the results cannot be transmitted to clinical outcome parameters without restrictions. Only few studies investigated the correlation between patients’ clinical situation and radiological findings with special interest of instability criteria after decompression. Some authors did not find any influence of the extent of the removed spinal structures on segmental stability or on clinical results [5–7]. In contrast, in other publications a positive coherency between signs of intervertebral instability and poor clinical outcome was described [8, 9]. Radiographic data have always been collected by the means of conventional x-rays in neutral position or in ante-/retroflexion. This is the standard procedure, which is known to be a relevant radiation exposure for the patient. It is not clear to date, whether these radiographs can be replaced or augmented by MRI (magnetic resonance imaging) in an upright position.

The purpose of this prospective consecutive study was to evaluate the clinical outcome in patients who underwent microsurgical decompression in lumbar spine and particularly to analyze segmental movement in the operated levels by use of upright, kinetic-positional MRI over a period of 12 months and then to correlate the clinical and imaging data with each other.

## Methods

This prospective, consecutive single-centre study was approved by the local ethical committee of Hannover Medical School (no. 2930–2015) and performed in accordance to the valid guidelines and regulations after obtaining informed consents from all participants.

### Patient population and inclusion criteria

According to biometrical sample size analysis a minimum number of 23 patients was required for statistical analysis (assumptions: paired t-test, two-sided, expected effect size dz = 0.79, alpha = 0.05, power = 0.95). To compensate for potential loss-to-follow-up a total of 30 patients were included in the study. All participants had indication for microsurgical decompression in the lumbar spine because of spinal canal stenosis with nerve root compression due to recess stenosis caused by facet joint arthrosis and hypertrophy of the yellow ligament and in some times additional degenerative spondylolisthesis. In patients with an additional disc prolapse detected on preoperative imaging (MRI or CT, computed tomography) sequestrectomy or diskectomy was planned to be performed as well. Patients with foraminal stenosis or extraforaminal (lateral) disc prolapse were not included in this study to ensure an identical surgical procedure during decompression.

### Surgical technique and postoperative therapy

All operations were performed by the same surgeon (DD) under microscopic view in one or two levels with approach to the spinal canal from one side to perform decompression either unilaterally or bilaterally in over the top-technique. Alternatively, an approach from both sides was also possible for bilateral decompressive procedure. The steps of the operation are standardized and consisted of laminotomy with removal of the lower part of the cranial lamina and the upper part of the caudal lamina as much as necessary, medial facetectomy and resection of the ligamentum flavum. In each case the medial structures of the dorsal elements of the spinal canal which were supra- and interspinous ligaments and spinous processes had not been removed and therefore were left completely intact. This procedure should prevent stability and minimize the risk for postoperative hypermobility in bilateral decompression of the spinal canal in comparison to the unilateral approach. In case of disk herniation the prolapsed disk material was also removed as spare as possible. Surgery had been finished when all stenosing elements were eliminated and the dural sac and nerve roots were completely free of any compression.

All patients were mobilized on the first postoperative day without an orthosis and they were advised only to take physical restraint for 6 weeks without any further restrictions.

### Clinical data

After inclusion in the study and giving their informed consent all participants got questionnaires with visual analogue scale (VAS) for back and leg, Oswestry Disability Index (ODI), Roland-Morris Disability Questionnaire (RMDQ) and Short-Form-36 (SF-36) with assessment preoperatively (*t0*) and after 6 weeks (*t1*) and 12 months (*t2*) [10–12]. Walking distance, analgesics according to classification of World Health Organization (WHO) into group I (non-opioid analgesics), II (low potency analgesics) and III (highly potent analgesics) and all complications within follow-up period were also documented.

### Measurements on upright, kinetic-positional MRI

**Fig. 1:**
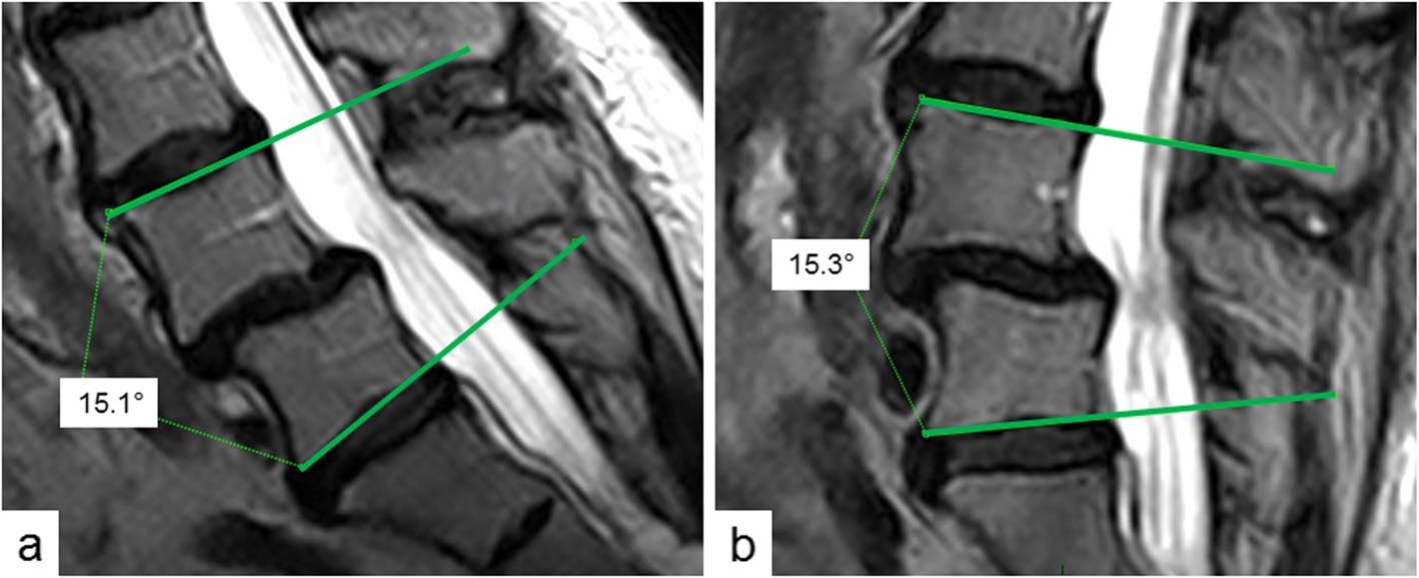
Measurement of intersegmental angle L4/5 in sitting position in correspondent median sections of the spine in Cobb’s technique in anteflexion (**a**) and retroflexion (**b**) [13]

**Fig. 2:**
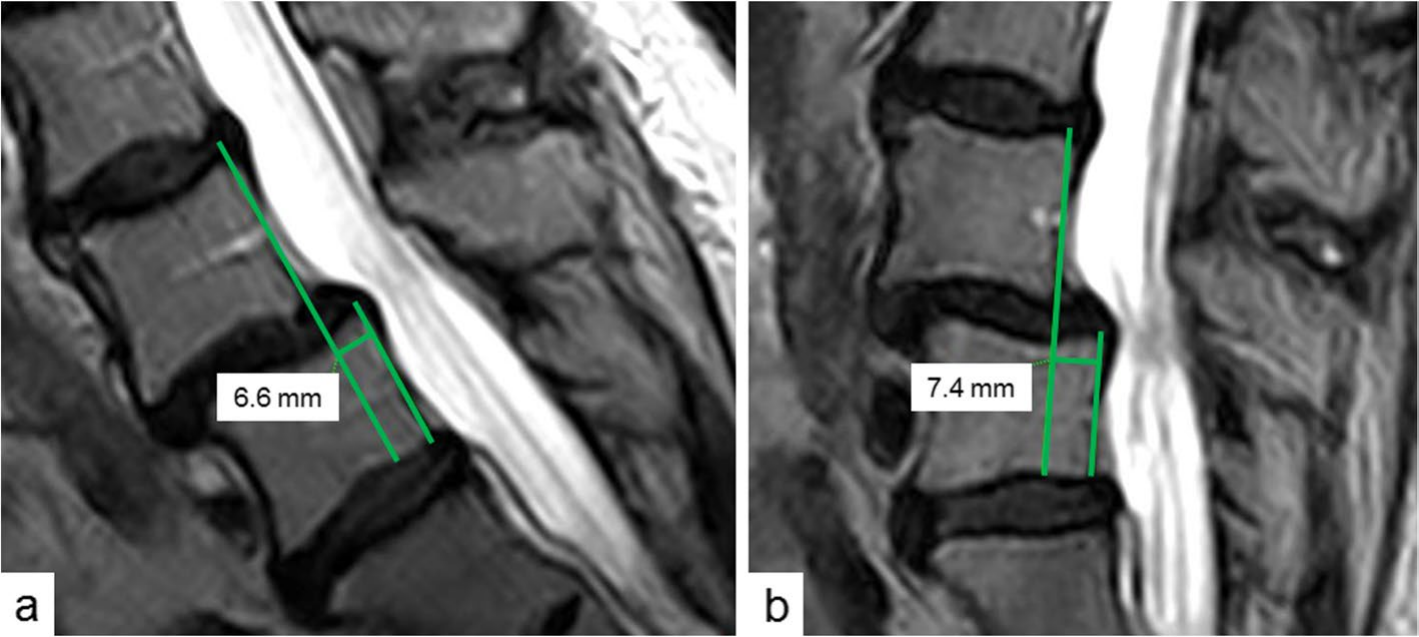
Measurement of translation L4/5 in sitting position in correspondent median sections of the spine in technique of Dupuis et al. in anteflexion (**a**) and retroflexion (**b**) [14]

The patients had upright, kinetic-positional MRI (FONAR Upright™ MRI, 0.6 T; FONAR Corporation; Melville; NY 11747, USA) before surgery (*t0*) and 6 weeks (*t1*) and 12 months (*t2*) postoperatively. All measurements were performed on sagittal T2-weighted images in ante- and retroflexion during the sitting position, i. e. axially weight-loaded. The participants were always asked to flex and extend to the most possible degree which was individually dependent on their range of motion and any occurrence of pain. After taking up the postures support rests were placed to maintain these positions each in in- and reclination during the scans. Intervertebral movement of the operated levels was determined in standardized technique on identical layers of the pictures always in the median section of the spine using the imaging software PACS 11 @XenApp (version 11.4.1.1011 – sias110, ©Carestream Health, Inc. 2011). Two methods were used for analysis of segmental movement. First, the intersegmental angles were measured according to Cobb’s recommendation (Fig. 1) [13]. The differences of the values in ante- and retroflexion in degree [°] were calculated as range of motion for statistical analysis. Second, translation (= horizontal displacement) between two vertebral bodies was measured in case of degenerative spondylolisthesis in the technique of Dupuis et al. (Fig. 2) [14]. Again, the differences of the values in ante- and retroflexion were collected for statistics with definition of instability by Dupuis et al. when displaying translation of more than 4 mm. All measurements were assessed by three independent, blinded examiners with determination of intraclass correlation coefficient (ICC).

### Statistical analysis

Wilcoxon-signed-rank-test for paired samples was used to calculate the *p*-value between all continuous variables at the three examination points of time (*t0*, *t1* and *t2*). Additionally, regression analysis by fitting a linear model between clinical scores and imaging data were performed. The coefficient of determination (R^2^) was used to determine the goodness-of-fit of the regression. All analysis were performed in R (version 3.5.0) with RStudio.

## Results

### Patient characteristics and surgical procedures

Out of the 30 consecutively included patients 24 participants had complete documentation on each of the three time points. The other 6 patients had stopped their controls by themselves due to personal, medical or professional reasons, so that the data of the sample of 24 patients were evaluated, statistically analyzed and presented in the subsequent section. 13 patients were female (54.2%) and 11 male (45.8%), the average age was 64.2 years (38.4 – 82.6 years). The microsurgical operations can be classified as follows:

- Number of levels:
  - One: 21
  - Two: 3
  - Total: 27
- Operated segments:
  - L2/3: 0
  - L3/4: 6
  - L4/5: 15
  - L5/S1: 6
- Decompression only:
  - Unilateral: 14
  - Bilateral from unilateral (over the top): 5
  - Bilateral from bilateral: 1
- Decompression plus sequestrectomy or diskectomy: 7

In all decompressive procedures the medial posterior elements of the spinal canal were left completely intact.

### Clinical outcome

**Fig. 3:**
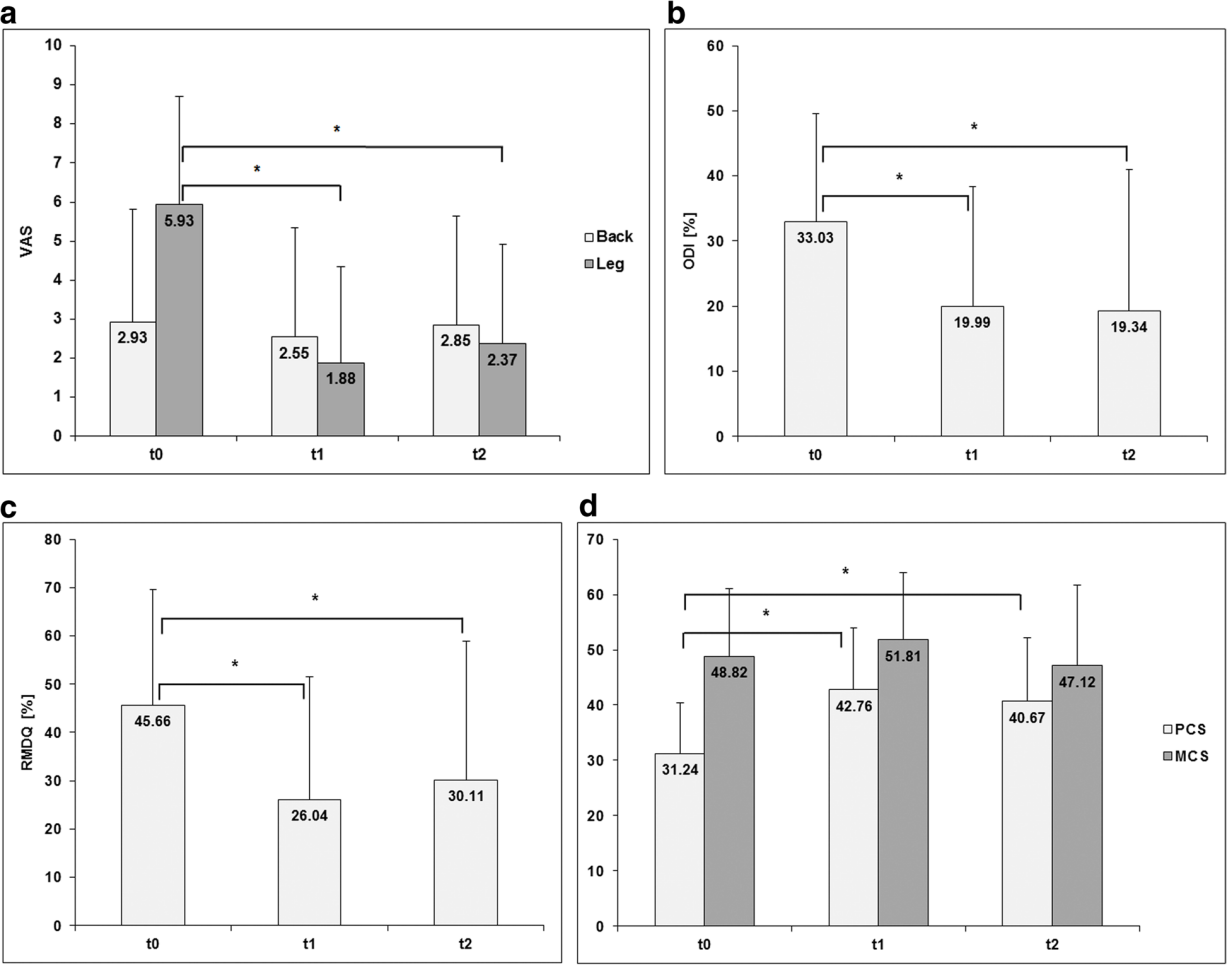
Clinical outcome over follow-up period, VAS back and leg (**a**), ODI (**b**), RMDQ (**c**), SF-36 (**d**). * indicates statistical significant difference

The intensity for leg pain was statistically significantly reduced at 6 weeks (*p* < 0.001) and 12 months (*p* < 0.001) postoperatively whereas VAS for back pain did not show any significant differences during the follow-up period (*t0* to *t1 p* = 0.330, *t0* to *t2 p* = 0.961) (Fig. 3a).

Functional impairment expressed in ODI and RMDQ also had been statistically significantly decreased at both controls (ODI: *t0* to *t1 p* = 0.003, *t0* to *t2 p* = 0.004; RMDQ: *t0* to *t1 p* = 0.001, *t0* to *t2 p* = 0.026) (Fig.3b, c).

Health-related quality of life according to SF-36 improved significantly in physical component scale (PCS) over time (*t0* to *t1 p* = 0.001, *t0* to *t2 p* = 0.016) with no significant changes in mental component scale (MCS) (*t0* to *t1 p* = 0.375, *t0* to *t2 p* = 0.603) (Fig. 3d).

Before operation 19 of the 24 patients had typical claudication with limitation in their walking distance with an average of 458 ± 389 m. After 6 weeks only 10 participants still reported a reduction of walking distance with a mean of 980 ± 763 m and at final follow-up just 8 persons had decrease of walking distance with an average of 1167 ± 1465 m.

Table 1 illustrates the numbers of patients with use of analgesics and with classification according to WHO at *t0*, *t1* and *t2*.

**Table 1:** Numbers of patients with use of analgesics and with classification according to WHO at *t0*, *t1* and *t2*

Date	*N*	WHO I	WHO II	WHO III
*t0*	18	22	2	5
*t1*	10	11	3	2
*t2*	12	14	2	3

### Complications and revision surgeries

In one patient incidental durotomy had occured with watertight closure and further uneventful course. Two participants suffered from recurrent disc prolapses after 9 months with subsequent microsurgical removal. In one patient a recurrency of a facet joint cyst became evident 5 months postoperatively with following resection. No patient had to undergo fusion surgery until the end of the observation period.

### Radiological results

**Fig. 4:**
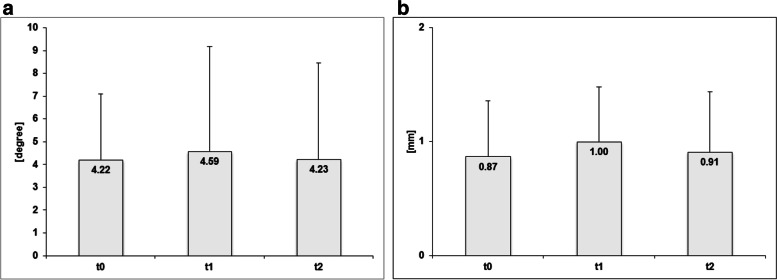
Radiologic findings during observation period with average differences of intersegmental angles of the 27 operated levels in degree (**a**) and average differences of translation in millimeters [mm] in the 13 levels with preoperative degenerative spondylolisthesis (**b**)

The average differences of the intersegmental angles in ante-/retroflexion of the 27 operated levels were not statistically significantly different during follow-up (*t0* to *t1 p* = 0.501, *t0* to *t2 p* = 0.972, *t1* to *t2 p* = 0.594) (Fig. 4a). ICC was 0.65 (95% confidence interval, CI: 0.55—0.75).

Translation between two adjacent vertebrae before surgical intervention was seen in 13 of the 27 operated segments (48.1%). According to Meyerding vertebral slippage was grade I in 11 and grade I to II in 2 patients, which means always slight spondylolisthesis. The mean differences of translation in ante-/retroflexion also showed no statistically significant differences at the three control dates (*t0* to *t1 p* = 0.402, *t0* to *t2 p* = 0.944, *t1* to *t2 p* = 0.588) (Fig. 4b). ICC was 0.49 (95% CI: 0.36—0.62). An instability according to definition of Dupuis et al. was not detectable at any point of time [14].

### Correlation between radiological and clinical results

**Fig. 5:**
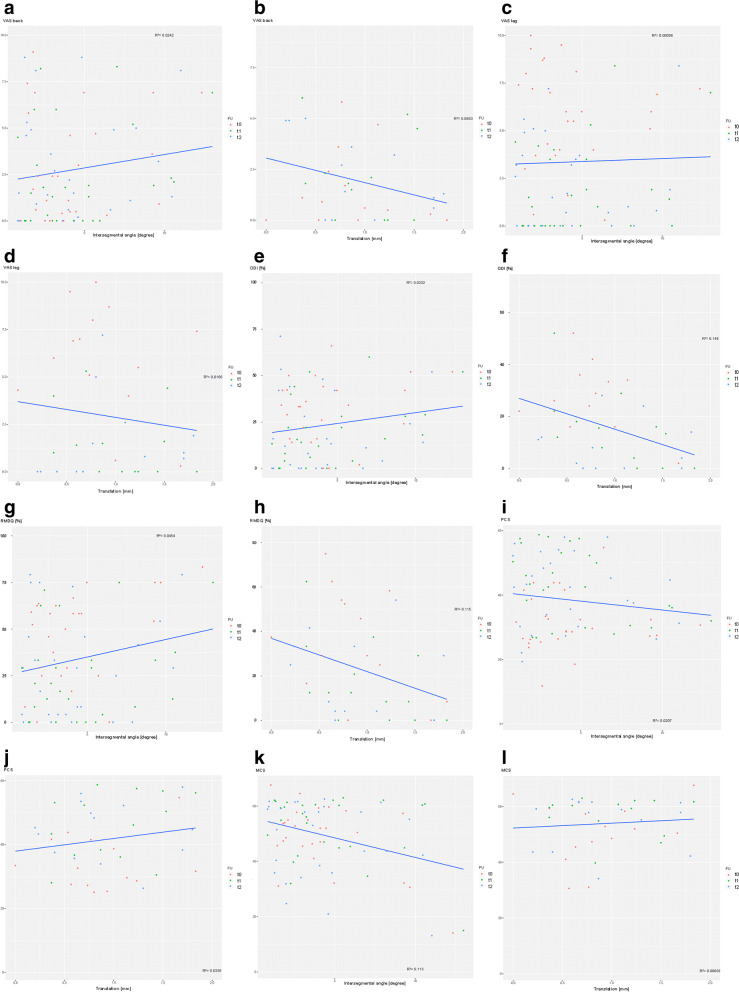
Regression analysis between the clinical scores and imaging parameters (intersegmental angle *n* = 24 patients; translation *n* = 13 patients): VAS back and intersegmental angle (**a**), VAS back and translation (**b**), VAS leg and intersegmental angle (**c**), VAS leg and translation (**d**), ODI and intersegmental angle (**e**), ODI and translation (**f**), RMDQ and intersegmental angle (**g**), RMDQ and translation (**h**), PCS of SF-36 and intersegmental angle (**i**), PCS of SF-36 and translation (**j**), MCS of SF-36 and intersegmental angle (**k**), MCS of SF-36 and translation (**l**)

Regression analysis was performed to find any dependencies between the clinical scores (VAS back and leg, ODI, RMDQ, PCS and MCS of SF-36) and imaging parameters (intersegmental Cobb angles and translation) (Fig. 5). In none of the evaluated associations could a linear dependency be verified.

## Discussion

In our study we investigated the clinical and imaging findings in a prospective, consecutive series of patients who had microsurgical decompression in the lumbar spine in a standardized way. The results of the 24 participants during follow-up period were positive with decrease in leg pain and improvement in functional outcome scores (ODI and RMDQ) as well as in physical component scale of SF-36 each with statistical significance. Furthermore, number of patients with limitation in walking distance and use of analgesics could be clearly reduced. These clinical outcome parameters confirm the correct indication for operation and are comparable to similar investigations [15–19]. The relatively low back pain intensity pre- and postoperatively can be explained by the underlying pathology, which was nerve root compression predominantly causing leg pain rather than dorsalgia. Moreover, the constant back pain levels over time could indicate maintaining intervertebral stability of the operated segments.

One focus of our work was analysis of the intersegmental motion of the 27 microsurgically decompressed levels. Upright, kinetic-positional MRI was used as diagnostic tool that is in contrast to similar studies in which conventional x-rays in neutral position or in flexion–extension had been performed [5–7, 9]. Only few publications exist dealing with analysis of movement of the spine by upright-MRI [20–22]. According to the authors‘ knowledge just one study evaluated a postsurgical effect which was after insertion of an interspinous distraction device [23]. More publications about upright-MRI focus on imaging analysis of spinal degeneration with emphasizing that kinetic-positional MRI is more specific and sensitive than conventional MRI and therefore effective for diagnosing, evaluating, and managing degenerative spine disease [24]. In more recent studies the authors also pointed out the relevance of MRI in upright-technique in patients with spinal canal stenosis with demonstration of clear dependence on body position [25, 26]. The main advantage of upright, kinetic-positional MRI is the possibility to simulate axial load on the spinal segments under weight-bearing which also can be applied in different body positions (neutral, ante- and retroflexion) for motion studies and therefore simulating true-to-life condition under avoidance of radiation exposure for the patients. Furthermore, people with claustrophobia can undergo MRI in most cases because of the open and more spacious examination unit.

Our imaging data measurements showed clear results with exclusion of any postsurgical increase in segmental motion or even in development of instability in the decompressed lumbar levels during follow-up period over 12 months. The chosen parameters were intervertebral Cobb angles and translation in the definition by Dupuis et al. to analyze intersegmental movement [13, 14]. No statistically significant differences between the control appointments were detected by the three independent, blinded examiners. The fair to good interobserver consistency strengthens the power of these results. The findings also represent no difference in uni- versus bilateral decompression technique and showed no influence on the segmental stability in case of additional removal of prolapsed disk material. Even in preexisting spondylolisthesis we observed no statistically significant change in intervertebral motion or translation meaning excluding progression of horizontal displacement. Similar results were published by some authors who also did not notice a significant influence of the radicalness of decompression and the clinical or radiographic results [5–7]. For example, Jalil et al. concluded that stability of the lumbar spine could even be maintained five years after interspinous, bilateral microsurgical decompression with resection of supra- and interspinous ligaments [6].

Regarding regression analysis between clinical scores and imaging data no dependence could be detected over entire follow-up period at all. This could imply that the intervertebral movement parameters either generally do not have any influence on clinical outcome or that the magnitude of the degree of intersegmental motion did not reach relevant extent in this study. In contrast, Kotilainen and Valtonen did observe clinical signs of symptoms of lumbar spinal segmental instability in 22% of their patients after a mean of three years after microdiskectomy with significant association between postoperative instability and unsatisfactory long-term outcome [8]. Similar findings were published by Schaller who described a positive correlation between the degree of extent of intervertebral disk resection and an increased risk of subsequent segmental instability and secondary deterioration of clinical and neural symptoms which had occurred in 0.5% of the patients after an average of 24 months postoperatively [9].

### Limitations

The authors are aware of some limitations of their study. One restriction might be first the relatively small number of participants. However, the results were unambiguous with no detection of postoperative intersegmental movement increase or even instability at all. Therefore, it can be assumed that the results were not essentially different if more patients would had been included. Second, microsurgical operation was mainly performed to decompress the neural structures exclusively on one side (21 out of 27), therefore resulting in an unequal distribution of uni- and bilateral spinal canal decompression which hints at a selection bias and should be considered when interpreting the results. Because of complete preservation of the posterior central structures of the spinal canal in all decompressive procedures (uni- and bilaterally) and the fact, that only one level had been operated bilaterally from both sides (the other five in over the top-technique from one side), we finally can suppose that the influence of the type of decompression maybe does not play a significant role in interpretation of the clinical and radiological results. Third, in almost a quarter (7 out of 27) of the operated levels a disk prolapse had been additionally removed, which could have had influence on intervertebral motion. In spite of the slight differences in surgical techniques the findings were completely consistent with no spikes in data after statistical analysis. Finally, the observation period had been finished after 12 months. Therefore, we cannot present long-term results and are unable to assess if our findings would be consistent during longer follow-up interval. However, even if segmental instability would be observed afterwards, it would be difficult to distinguish if it is still a direct effect of postsurgical alteration or just a degenerative change due to natural course.

## Conclusions

In this prospective consecutive patient series microsurgical decompression of the lumbar spine did not lead to increase of intersegmental movement or even instability and the results were independent on condition of preexisting spondylolisthesis or on some variations in operative technique (uni- versus bilateral decompression, additional disk prolapse removal) over a follow-up period of 12 months. Furthermore, the magnitude of intervertebral motion showed no correlation to the clinical score parameters at all three examination points of time.

Because of the limitations of the study as mentioned above a series with a higher number of patients and with a longer follow-up interval could give more information about the characteristics of intersegmental movement of the lumbar spine after different decompressive surgical techniques.

## Data Availability

The datasets used and/or analysed during the current study are available from the corresponding author on reasonable request.

## Abbreviations

MRI: Magnetic Resonance Imaging
VAS: Visual Analogue Scale
ODI: Oswestry Disability Index
RMDQ: Roland-Morris Disability Questionnaire (RMDQ)
SF-36: Short-Form-36
CT: Computed Tomography
WHO: World Health Organization
ICC: Intraclass Correlation Coefficient
CI: Confidence Interval
